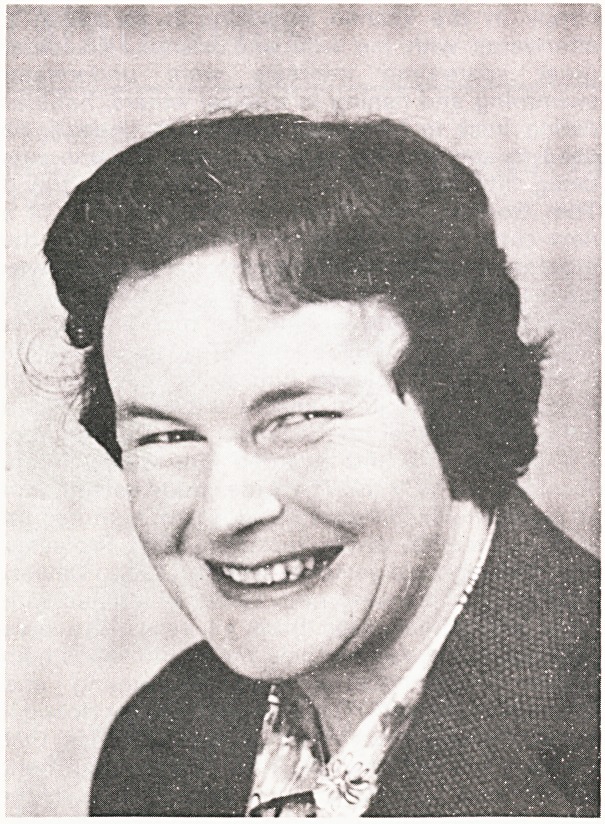# Dr. Kathleen Iles

**Published:** 1980

**Authors:** 


### Dr. Kathleen Constance Iles M.B., Ch.B.

Dr. Kathleen Iles, who was Medical Assistant at Ham Green Hospital and the Bristol Chest Clinic, died in her sleep on 9th October 1980 at the age of 60 years.

Kay, as she was known to all, was born on 26th December 1919, in Clifton, the daughter of Ernest Arthur Iles, OBE, F.R.C.S., who was for many years Consultant Surgeon to Bristol Eye Hospital and was also Honorary Treasurer of Bristol Medico-Chirurgical Society. He and Kay's mother had met whilst serving in H.M. Hospital Ship 'Plassey' during the first World War.

Kay was educated at Clifton High School and started her medical training in the University of Bristol where she was a keen oarswoman and tennis and squash player. In 1943 at the age of 24 years she was found to have Pulmonary Tuberculosis. This was long before the days of modern chemotherapy and Kay experienced the full terrors of the old methods of treatment culminating in a 2-stage Thoracoplasty. After much patience and great courage she eventually won the battle but it was three years before she was able to resume her medical studies and eventually qualify in 1948.

Her career in medicine lay inevitably in the realms of Diseases of the Chest and after experience at Winsley and Ventnor Sanatoria she returned to Bristol in 1954 as Medical Registrar and then as Medical Assistant to Ham Green Hospital and the Bristol Chest Clinic. For over a quarter of a century Kay was the key member of the Bristol Chest Team – an essential link between all her medical and surgical and nursing colleagues and their patients. She was in every way the perfect Medical Assistant, loyal and hard-working and completely dependable and capable of dealing calmly and effectively with every crisis, whether it were medical or emotional or administrative. Although easily short of breath and often tired she never complained and never showed an inkling of self-pity. Her own experiences gave her a deep and sympathetic understanding of the problems and reactions of her patients who admired her greatly and trusted her implicitly.

Kay had a tremendous regard for the Junior Medical Staff and many a young Pre-Registration House Physician will be the better doctor today because of her wise and patient instruction and the example she set of devoted and conscientious work. Her service to Ham Green Hospital was not confined to her medical work but extended enthusiastically into the social life of the Hospital and not least to the skittles team of which she was an energetic member. Her interests outside medicine were wide and she had a great love of nature and music and literature. She was an enthusiastic photographer, and accomplished cook, a skilful seamstress and an avid follower of tennis. Not only her father but her brother, Dick, her step-mother Anna and her sister Ann were all doctors and she was never happier than when she was called to 'sit-in' for Ann's children. She was loved and admired by all who knew her and will be greatly missed by her family and by all her friends and colleagues and in the day-to-day life of Ham Green Hospital.

A.T.M.R.

## Figures and Tables

**Figure f1:**